# Determination of the UV Inactivation Constant Under 280 Nm UV LED Irradiation for SARS‐CoV‐2

**DOI:** 10.1111/php.13653

**Published:** 2022-06-17

**Authors:** Biffi Silvia, Signorini Lucia, Cattaneo Luciano, Della Corna Lorenzo, Guercilena Andrea, D’Alessandro Sarah, Ferrante Pasquale, Delbue Serena

**Affiliations:** ^1^ Light and Colour Engineering s.r.l Piazza Della Repubblica Mediglia Italy; ^2^ Laboratory of Molecular Virology, Department of Biomedical, Surgical and Dental Sciences University of Milan Milan Italy; ^3^ Mireide Electronics s.r.l. Lodi Italy; ^4^ Simaco Elettromeccanica s.r.l. Lodi Italy

## Abstract

The ongoing emergency provoked by the SARS‐CoV‐2 pandemic demands the development of technologies to mitigate the spread of infection, and UV irradiation is a technique that can efficiently address this issue. However, proper use of UV equipment for disinfection requires an understanding of how the effects on SARS‐CoV‐2 are dependent on certain parameters. In this work, we determined the UV‐C inactivation constant k for SARS‐CoV‐2 using an LED source at *λ* = 280 nm. Specifically, a Log3 reduction was measured after irradiation for 24 min with a delivered UV‐C dose of 23 J m^−2^. By multitarget model fitting, *n* = 2 and k = 0.32 ± 0.02 m^2^ J^−1^ were obtained. A lag time for the inactivation effect was also observed, which was attributed to the low irradiation levels used to perform the study. The combination of k and delay time allows for reliable estimation of disinfection times in small, closed environments.

## Graphical abstract

The ongoing SARS‐CoV‐2 sanitary emergency demands the development of technologies to mitigate the spread of infection. UV irradiation can address this purpose. UV‐C inactivation constant k was determined using an LED source at *ʎ* = 280 nm. A Log3 reduction was measured after irradiation for 24 min (delivered UV‐C dose of 23 J m^**−**2^). By fitting with the multitarget model, *n* = 2 and k = 0.32 ± 0.02 m^2^ J^**−**1^ were obtained. A lag time in the inactivation effect was also observed, which was attributed to the low irradiation levels used. The combination of k and delay time allows reliable estimation of disinfection times in any environment.

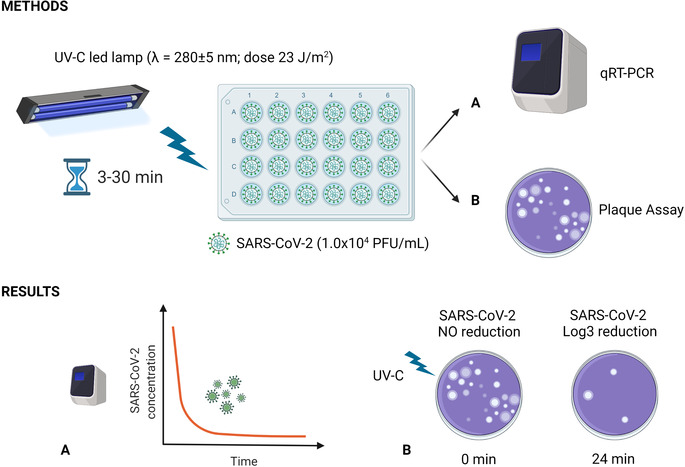

**Abbreviations**
BSL‐3: biosafety level‐3
CPEs: cytopathic effects
DMEM: dulbecco's modified eagle's medium
DNA: deoxyribonucleic acid
DPBS: dulbecco's phosphate‐buffered saline
FBS: fetal bovine serum
LED: light emitting diode
min: minutes
MOI: multiplicity of infection
NSP: nasal pharyngeal swab
PFU: plaque forming unit
qRT–PCR: quantitative reverse transcriptase‐PCR
RNA: ribonucleic acid
SARS‐CoV‐2: severe acute respiratory syndrome‐coronavirus‐2
t: time
UV: Ultraviolet

## INTRODUCTION

The recent emergency originating in 2020 by the spread of Severe Acute Respiratory Syndrome Coronavirus‐2 (SARS‐CoV‐2) infection has increased attention on disinfection methods to prevent infections and reduce the spread of the virus.

In particular, Ultra Violet (UV) light irradiation has received increasing attention since it is a well‐established, simple, effective, noncontact method to inactivate pathogenic microorganisms, such as viruses, bacteria and spores (1). UV irradiation can be efficiently used to inactivate viruses in saliva droplet suspensions floating in closed environments or deposited over surfaces or equipment (2).

The UV light spectrum covers the range of 100–400 nm and is divided into UV‐A (400–315 nm), UV‐B (315–280 nm) and UV‐C (100–280 nm) regions. Light from the UV‐C and UV‐B regions can be absorbed by DNA, RNA and proteins of microorganisms, causing alterations in their molecular structures and thereby affecting their replication process. The efficiency of UV exposure depends on the wavelength, with a maximum efficiency at approximately 260–265 nm. The structure of microorganisms also plays a role, so the actual efficiency may vary between species (1).

Microorganisms irradiated with UV light are exposed to a dose *D* = I*t (J m^**−**2^), which is a function of irradiance *I* (W m^**−**2^) and exposure time *t* (s). The fraction of survival, *i.e*. the fraction of microorganisms still active after irradiation time t, can be described as *F* = exp(−k*D), where *k* is the UV rate constant (m^2^ J^**−**1^) and is species‐ and wavelength‐dependent. *k* represents the susceptibility of a particular pathogen to UV exposure: high values of *k* are associated with fast decay and thus fast disinfection, while low values of k are associated with slow decay and longer disinfection times (2).

A review of the effects of UV‐C light on coronaviruses was reported by Kowalski *et al*. (3). Recently, many works have focused specifically on SARS‐CoV‐2, reporting that significant inactivation can be successfully achieved using UV‐C light (4, 5, 6, 7, 8, 9, 10). In particular, Minamikawa *et al*. used three Light Emitting Diode (LED) light sources exhibiting peaks at *λ* = 265, *λ* = 280 and *λ* = 300 nm and found that Log3 inactivation was achieved when the virus was exposed to doses of 18, 30 and 230 J m^**−**2^, respectively; the effects of wavelength on SARS‐CoV‐2 inactivation were also measured (9). LEDs with a peak at 280 nm are a solid technology commercially available at low cost and with stable emission over time. For these reasons, *λ* = 280 ± 5 nm LEDs are ideal for developing new low‐cost devices. In this work, we focused on the interaction between SARS‐CoV‐2 under wet conditions using viral particles suspended in liquid media and UV‐C light irradiation at *λ* = 280 ± 5 nm by measuring in detail the inactivation of SARS‐CoV‐2 over time and determining its UV‐C inactivation constant k. By using extremely low irradiation values, which resemble working conditions for UV‐C disinfection in small, closed environments, we studied for the first time the early stages of SARS‐CoV‐2 inactivation and observed a lag time in the response of the inactivation process.

## MATERIALS AND METHODS

### UV illumination system

The light source used in this study was a modified version of a commercial lamp (KATARI®) built and marketed by Simaco Elettromeccanica, Italy. Simaco supplied a lamp that was modified with respect to those that the company builds and markets. The lamp was depowered to allow low irradiance levels in small laboratory environments, such as hoods. Simaco also supplied a screening device to contain UV‐C light and a commercial power meter (Spectroradiometer—JEDI Technische Instrumente specbos 1211 UV‐2‐LAN) to measure irradiance on site. The lamp was equipped with three UV‐C LEDs emitting in the range of *λ* = 280 ± 5 nm (Figure S1) and with aluminum reflectors that modified the beam in a controlled way, enabling increased radiation emission levels and improved uniformity on the target. The distance between the light source and the samples must be almost 10 times the linear dimension of the source module (or the sensor) to avoid reading errors due to the cosine law followed by sensor devices (11). Given the geometrical characteristics of the lamp, the samples were placed at a distance of 0.57 m from the lamp. Calculations were performed for incident irradiance, with distance measured from the fluid surface. Irradiance on the samples was measured to be *I* = 0.182 W m^**−**2^. At such low irradiation values, it was possible to control the UV‐C doses delivered to the samples through exposure time with negligible experimental error. Exposure times were chosen in the range of 0–30 min. In three independent experiments, irradiation was performed for *t* = 3, 6, 12 and 24 min, while a single experiment was performed with irradiation for *t* = 3, 7.5, 15 and 30 min.

### Isolation of SARS‐CoV‐2 from nasopharyngeal swab

Nasal pharyngeal swab (NPS) was collected upon approval of the Local Ethical Committee and signature of the informed consent (Fondazione Ca′ Granda, Ospedale Maggiore, Milano, Italy approved the protocol 456_2020, on May 2020). SARS‐CoV‐2 was isolated from 500 μL of NSP of a COVID‐19 patient, added to Vero cells (ATCC CCL‐81), and maintained in complete medium composed of Dulbecco's modified Eagle's medium (DMEM) high glucose containing 10% heat‐inactivated fetal bovine serum (FBS), 2 mM L‐glutamine and antibiotics (Euroclone, Italy) at 80% confluence; the inoculum was removed after a 3‐h incubation at 37°C with 5% CO_2_, and the cells were incubated at 37°C and 5% CO_2_ for 72 h, when cytopathic effects (CPEs) were evident. Isolation of the virus was confirmed by specific quantitative reverse transcriptase‐PCR (qRT–PCR) (12), which targets the N1 region of the SARS‐CoV‐2 nucleocapsid gene, and by complete genome sequencing, as previously described (13). The isolated strain was subsequently titrated by plaque assay using dilution factors ranging from 10^1^ to 10^9^ and was used at a multiplicity of infection (MOI) of 0.01 in subsequent experiments. The complete nucleotide sequence of the isolated SARS‐CoV‐2 strain was deposited at GenBank at NCBI (accession number: MT748758.1).

### SARS‐CoV‐2 irradiation

Before irradiation, the viral stock was diluted in DMEM high glucose with sodium pyruvate, without L‐glutamine, 2 mm L‐glutamine, and 1X penicillin and streptomycin. Then, 60 μL of this solution (10 000 Plaque Forming Unit ‐PFU‐ mL^**−**1^) was added in triplicate to a 24‐well plate. Medium without virus (mock) was used as a control. The light source was placed over the plate at a height of 0.57 m. Irradiation was performed for 3, 6, 7.5, 12, 15, 24 and 30 min (*N*). A copy plate with complete medium containing the virus was subjected to the same irradiation steps with a regular light lamp (zero‐irradiation control, *N*_0_). The experiment was repeated three times.

### Evaluation of antiviral activity

The day before irradiation, Vero E6 (ATCC CRL‐1586) cells were seeded into 96‐well plates at a density of 1.3 × 10^4^ cells per well in complete medium and incubated at 37°C with 5% CO_2_. Immediately after irradiation following the SARS‐CoV‐2 irradiation scheme (3, 6, 7.5, 12, 15, 24 and 30 min of irradiation [*N*], zero‐irradiation control [*N*_0_] and mock), 30 μL of each viral suspension (MOI equivalent to 0.01) was added to Vero E6 cells, which were inoculated for 2 h at 37°C with 5% CO_2_. The viral inoculum was removed, the cells were washed with Dulbecco's phosphate‐buffered saline (DPBS), 200 μL of complete medium was added, and the plate was incubated at 37°C with 5% CO_2_. On day 2 postinfection, CPEs were evaluated under a light microscope. Culture medium was harvested, and RNA was isolated using the NucleoSpin RNA Virus kit (Macherey Nagel, Germany) following the manufacturers' protocol. Quantification of viral copy numbers was evaluated *via* qRT–PCR (12). Data are expressed as the fraction of residual activity, *F* = *N*/*N*_0_, where *N*_0_ and *N* represent the viral amount (SARS‐CoV‐2 copies mL^**−**1^) in the wells not subjected and subjected to irradiation, respectively.

### Evaluation of the virucidal activity by plaque assay

The virucidal effect of UV‐C irradiation was evaluated by means of a plaque assay performed on the infected cell medium harvested from the antiviral activity experiments. For each time point, performed in triplicate, supernatants obtained from the infected and irradiated cells were pooled into one data point. Moreover, 400 μL of each well of Vero E6 cells was added in duplicate and plated the day before in a 6‐well plate in complete medium (7.5 × 10^5^ cells per well). Briefly, after 2 h of inoculation with the pooled viral suspensions, the inocula were removed, and the cells were covered with a 0.3% agarose gel, dissolved in complete medium, and incubated for 48 h at 37°C with 5% CO_2_. Cells were then fixed with 4% formaldehyde solution and, after agarose removal, stained with 0.5% methylene blue. Plaques were counted, and the results are expressed as the fraction of residual activity, *F* = *N*/*N*_0_, where *N*_0_ and *N* represent the plaque number (PFU mL^**−**1^) obtained in the wells containing the medium of cells infected with zero irradiation and the irradiated samples, respectively.

All experiments were performed in a BioSafety Level‐3 (BSL‐3) laboratory.

## RESULTS

Cytopathic effects were clearly observed in SARS‐CoV‐2‐infected Vero E6 cells not subjected to irradiation (zero‐irradiation control), while the CPEs progressively decreased in infected cells subjected to an increased dose of radiation, whereby cell morphology was largely comparable to that of mock cells after ≥24 min of irradiation (data not shown).

**Figure 1:**
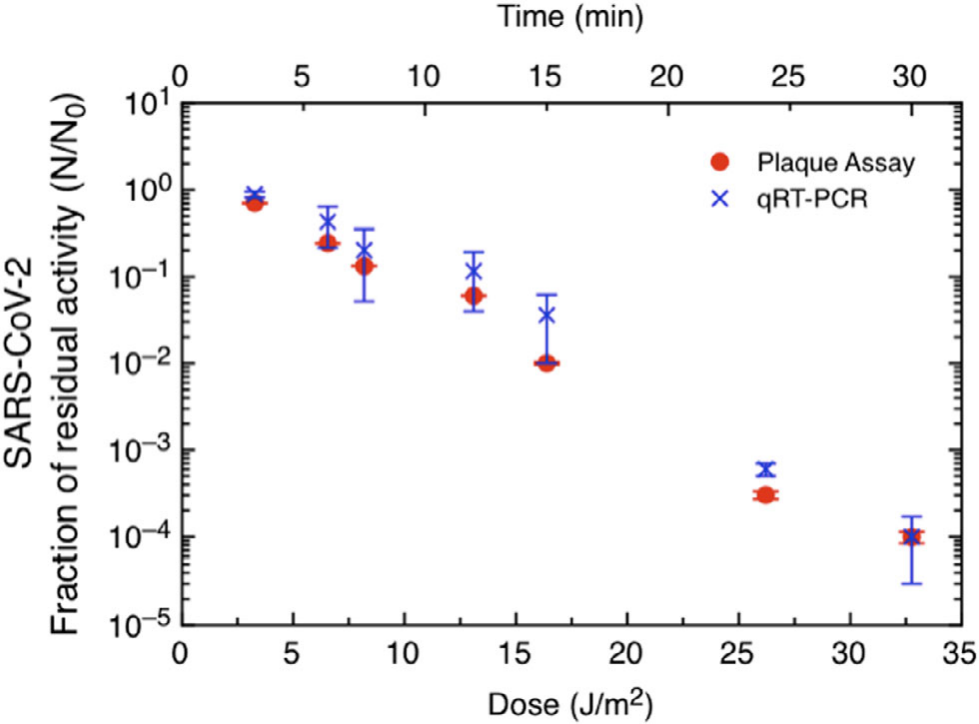
UV‐C‐irradiated SARS‐CoV‐2 (MOI 0.01) residual activity in Vero E6 cells *in vitro*. SARS‐CoV‐2 was irradiated for 3–30 min and then inoculated into Vero E6 cells, which were harvested 48 h postinfection. The residual activity of the virus was assessed by qRT–PCR (red dot) and plaque assay (blue stars). All experiments were conducted in triplicate.

**Figure 2:**
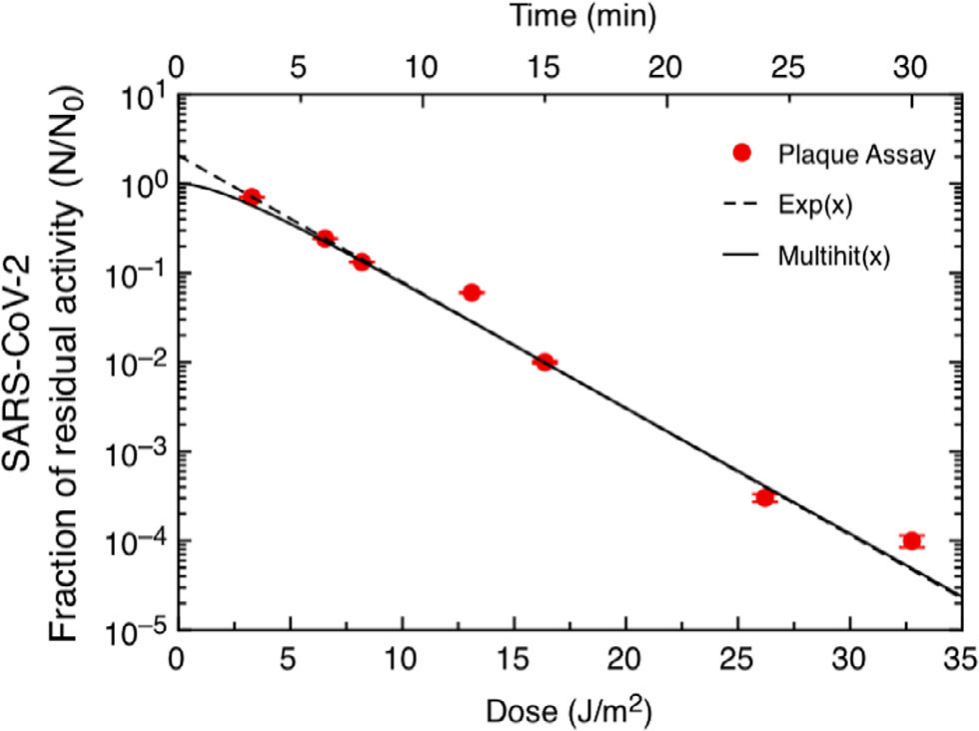
Data from the SARS‐CoV‐2 plaque assay on a semilogarithmic scale. Data are fitted with a single exponential model *F* = exp(−k*D) (dashed line) and with a multitarget model *F* = 1−[1−exp(−k*D)]^*n*^ (solid line).

**Figure 3:**
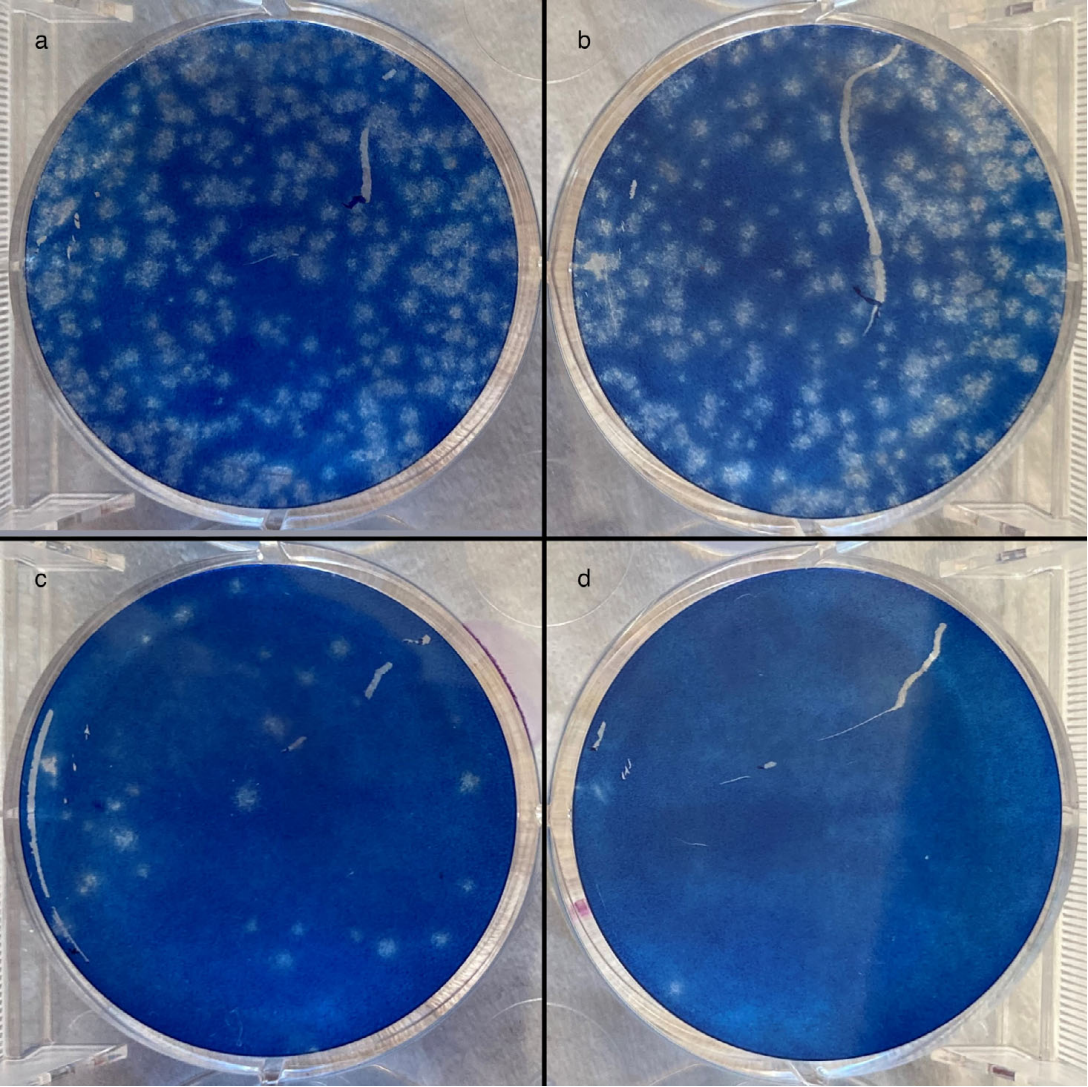
Plaque assays. Representative data from three independent assays after (a) no irradiation (0 min) and (b) 3 min, (c) 24 min and (d) 30 min of irradiation.

Viral activity after UV‐C exposure was measured by qRT–PCR and plaque assay techniques. The calculations for UV dose were made using the incident irradiance value and were not corrected for either absorbance or sample depth. The absorbance of the DMEM at 280 nm was measured and it was 5.3 cm^**−**1^ (Figure S2), while the sample depth was calculated to be 300 μm. The actual transmittance through the 300 μm was estimated to be 0.69 (69%). The data shown in Fig. 1 are expressed as a fraction of residual activity, *F* = *N*/*N*_0_, where *N*_0_ and *N* represent the viral amount before and after irradiation, respectively. As expected, the fraction of survival was significantly affected by UV‐C irradiation. The linear scale stresses the difference in significance of the two measurement techniques. qRT‐PCR (blue stars) measures the total amount of viral RNA recovered without distinguishing between infectious viruses and nucleic acids derived from inactivated viruses and thus provides a higher count, especially at low doses, *i.e*. low exposure times. In contrast, the plaque assay (red dots) quantifies the actual viral residual activity and is thus a more suitable indicator of the amount of infectious virus. Figure 2 shows the data obtained from the plaque assay on a semilogarithmic scale, and it can be appreciated that the viral activity decreases up to 10^−4^ in the time range explored. A Log_3_ reduction was measured after an irradiation time of 24 min and a delivered UV‐C dose of 23 J m^**−**2^. Representative data (irradiation for 0, 3, 24 and 30 min) from three independent plaque assays are shown in Fig. 3.

It is evident from Fig. 2 that the data can be approximated with a single exponential, but the value of the intercept at *D* = 0 is greater than one. Additional data should be collected to better define the early stages of inactivation, but in this case, the decay may be better approximated by a shoulder curve, starting out horizontally and developing a full exponential decay only after a few minutes of irradiation. Shoulder curves can be described by the multitarget model, which assumes that to inactivate a single microorganism, a critical number *n* of discrete sites has to be hit. The fraction of residual activity is then expressed as

(1) F=1−1−exp−k*D^n

where *n* is the multitarget exponent and is unique for each species (1, 2, 14). According to the multitarget model, the *n* value can be found by extrapolating the exponential stage data to the y‐intercept (2). Data were first fitted to a single exponential curve to extrapolate the intercept with the *y*‐axis. The value of the intercept was *y* = 2.0. The whole set of data was then fitted to Eq. (1), where *n* = 2.0 was imposed and the only free parameter left was *k*. As a result, the *k* value obtained was *k* = 0.32 ± 0.02 m^2^ J^**−**1^. In both cases, the Levenberg–Marquardt fitting algorithm was used.

## DISCUSSION

The literature on UV‐C inactivation of SARS‐CoV‐2 has mainly focused on working conditions characterized by relatively high irradiance and short distances between the light source and virus sample. This work has been extremely important to confirm the efficacy of UV‐C irradiation on SARS‐CoV‐2 and to establish the working conditions for its rapid and effective inactivation. The studies of Storm (7) and Biasin (8) were conducted using low‐pressure mercury lamps (254 nm) and irradiating the virus sample with 10.82 and 8.49 W m^**−**2,^ respectively; in the work of Minamikawa (9), deep ultraviolet light‐emitting diodes (265, 280 and 300 nm) were used, with effective irradiance values of 0.92, 0.83 and 9.25 W m^**−**2,^ respectively. In these works, the SARS‐CoV‐2 inactivation curves observed are well described by single exponential decays or, as in the work of Storm (7) under dry conditions, by a double exponential. The particularly low irradiance level used in the present study *I* = 0.182 W m^**−**2^, was chosen instead to mimic irradiation conditions that could be typical in UV‐C disinfection of small, closed environments, *e.g*. with UV‐C lamps set on the walls and ceilings of small environments. As a reference, with a lamp emitting 0.1 W in Lambertian mode placed 3.3 m high on the ceiling of an office, the irradiance on a desk (0.7 m high) would be on the order of 4.7 × 10^−3^ W m^**−**2^ on the surface perpendicular to the optical axes.

We found that when SARS‐CoV‐2 is treated with low‐intensity UV‐C light, the shape of its inactivation curve differs from that observed using high‐intensity irradiation. This behavior was unexpected and it needs to be explored. Previous works forced single‐stage decay through zero exposure concentration. We did not make such an assumption in our study, which led to the observation that there may be a shoulder associated with the inactivation of the virus. Future studies should include additional data collection in the region below the lowest UV‐C dose explored in this work to better describe the early‐stage behavior of virus inactivation. The shoulder curve reported in this work is in contrast to the recent observation that inactivation of SARS‐CoV‐2 under wet conditions is better described by a single decay (7). Such a difference in behavior can be attributed precisely to the different irradiation levels used in the two works. Given the low irradiation level used, the lag in response observed in this work can be explained by the presence of a threshold dose that has to be reached to provoke significant damage (1). Once the threshold dose has been reached, the inactivation process begins/progresses, and the single exponential decay is restored. Indeed, the value of the UV‐C inactivation constant determined here, *k* = 0.32 ± 0.02 m^2^ J^**−**1^, is consistent with that reported in the work of Minamikawa (9), where a *k* value of 0.30 m^2^ J^**−**1^ for *λ* = 280 nm was found.

The shoulder effect has also been observed in other studies on coronaviruses (15) and might be particularly important for the safe application of UV‐C disinfection in daily situations, which requires further investigation. The presence of this effect suggests that irradiation times for disinfection cannot be estimated solely based on extrapolation from measurements conducted at short distances but should consider the irradiation value actually delivered on a given surface, which diminishes as the square of the distance from the light source. According to Kowalski *et al*. (2), the amplitude of the shoulder, *i.e*. the delay in inactivation, is inversely proportional to the irradiation flux; thus, the delay would be increased on surfaces far from the UV‐C source.

Overall, we confirmed that UV‐C LED disinfection could be useful to increase the containment of SARS‐CoV‐2 spread, especially in medical environments and community settings, where devices equipped with UV‐C LEDs might be employed. It should also be noted that limited, accidental exposure to UV‐C LED light (*e.g*. accidentally stepping into a room where UV‐C LEDs are on) most likely will not exceed the “safety exposure level”, which is defined as 30 J m^**−**2^ by the guidelines of ICNIRP and IRPA/INIRC (16, 17).

The main limitation of this paper was the lack of a description of the *k* value and a quantitative description of the inactivation delay time under dry conditions *versus* wet conditions. Future studies in this direction would allow us to define a reliable estimate of disinfection times on different types of surfaces and environments. Moreover, it is necessary to better define the possibility of using UV‐C LED irradiation to inactivate SARS‐CoV‐2 in body fluids, such as saliva and/or respiratory samples that may be easily found on surfaces, and in aerosols composed of different sizes of microdroplets.

## CONFLICT OF INTERESTS

The authors declare no conflicts of interest. SIMACO Elettromeccanica s.r.l. did not influence the work or the results reported in this paper.

## AUTHOR CONTRIBUTIONS

L.C., L.D. and S.D. designed the study. L.S. and S.DA. performed the measurements. S.B., L.C., S.D. and P.F. analyzed the data. S.B., L.C. and S.D. wrote the paper.

## Supporting information


**Appendix S1** Supplementary materials.
