# Supplementary material for: Determination of the UV Inactivation Constant Under 280 Nm UV LED Irradiation for SARS‐CoV‐2
Source: Photochem Photobiol. 2022 Jun 17:10.1111/php.13653. Online ahead of print. doi: 10.1111/php.13653 (PMC9347497; doi:10.1111/php.13653)
Supplement: Supplementary file 1 — Appendix S1 Supplementary materials. [file PHP-9999-0-s001.docx]

**SUPPORTING INFORMATION**

**Determination of the UV Inactivation Constant under 280 nm UV LED Irradiation for SARS-CoV-2**

Biffi Silvia^1^*^†^, Signorini Lucia^2†^, Cattaneo Luciano^1^, Della Corna Lorenzo^3^, Guercilena Andrea^4^, D’Alessandro Sarah^2^, Ferrante Pasquale^2^, Delbue Serena^2^.

^1^Light and Colour Engineering s.r.l, Piazza Della Repubblica, 20060 Mediglia (Mi)

^2^Laboratory of Molecular Virology, Department of Biomedical, Surgical and Dental Sciences, University of Milan, Milan, Italy

^3^Mireide Electronics s.r.l., Via Solferino 55, 26834 Lodi (Lo)

^4^Simaco Elettromeccanica s.r.l., ex ss235 n.16, 26834 Lodi (Lo)

†These authors equally contributed to the project

*Corresponding author e-mail: [silvia.biffi@lcolor.it](mailto:silvia.biffi@lcolor.it) (Silvia Biffi)

**Materials and Methods**

Figure S1. LED emission profile of the employed lamp. The lamp was equipped with three UV-C LEDs emitting in the range of λ=280±5 nm

**Results**

Figure S2. Absorbance profile of DMEM. The measurement was performed over an optical path of 1mm. Absorbance is measured as A=-log_10_(I/I_0_).
